# Discovery of a Superconductor Bi_5_O_4_S_3_Cl Containing the Unique BiS_3_ Layer

**DOI:** 10.1002/advs.202303569

**Published:** 2023-08-27

**Authors:** Yaling Yang, Xiao Fan, Jiali Liu, Cheng Cao, Zhaolong Liu, Jun Deng, Ting Lin, Qinghua Zhang, Ke Liao, Xiaoli Dong, Gang Wang, Xiaolong Chen

**Affiliations:** ^1^ Beijing National Laboratory for Condensed Matter Physics Institute of Physics Chinese Academy of Sciences Beijing 100190 China; ^2^ University of Chinese Academy of Sciences Beijing 101408 China; ^3^ Songshan Lake Materials Laboratory Dongguan 523808 China

**Keywords:** BiS_2_‐based layered superconductors, BiS_3_ layer, coordination structure, superconductivity

## Abstract

The BiS_2_‐based layered superconductors with structures similar to those of cuprates and iron‐based superconductors have stimulated much research interest. Here, a new quaternary compound is reported, Bi_5_O_4_S_3_Cl, which crystalizes in a tetragonal structure with *P*4/*mmm* (No. 123) space group having alternately stacking unique BiS_3_ layers and Bi_2_O_2_ layers along the *c*‐axis with a Cl atom located at the center of the unit cell. A superconducting transition above 3 K is observed for both electrical transport and magnetic measurements. Hall resistivity measurements show its multiband character with a conduction dominated by electron‐like charge carriers. The first‐principles calculations exhibit that the semiconducting parent phase Bi_5_O_4_S_3_Cl becomes metallic when sulfur vacancies are introduced, which hints the origin of superconductivity in Bi_5_O_4_S_3_Cl. The findings will inspire the exploration of new BiS‐based superconductors.

## Graphical abstract

A new quaternary compound Bi_5_O_4_S_3_Cl, containing the unique BiS_3_ layer (in which Bi and S atoms form a six‐coordinated octahedron) and Bi_2_O_2_ layer with a Cl atom at the center of the unit cell, is discovered. A superconducting transition above 3 K is observed. This finding will shed light on the exploration of new BiS‐based superconductors.

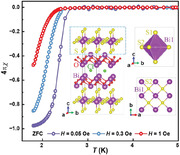

## 1 Introduction

Bismuth‐based layered materials have attracted extensive attention for their diverse functional layers and fascinating properties.^[^ ^1^ ^]^ For instance, Bi_2_Sr_2_CaCu_2_O_8_ (Bi2212) and Bi_2_Sr_2_Ca_2_Cu_3_O_10_ (Bi2223) are two representatives of high‐temperature cuprate superconductors with corresponding superconducting transition temperatures (*T_c_* s) of 85 K and 110 K, respectively.^[^ ^1a^ ^]^ Their structures are alternatively stacked by blocking layer (rocksalt‐like BiO and SrO layers) and different number of superconducting CuO_2_ layer (separated by calcium).^[^ ^2^ ^]^ BiCuSeO has been reported as a promising thermoelectric material for its intrinsic low lattice thermal conductivity, large Seebeck coefficient, and favorable crystal structure with an insulating Bi_2_O_2_ layer and a conductive Cu_2_Se_2_ layer.^[^ ^1^, ^3^ ^]^ The unique structure endows it with great potential for independently modulating the electrical and thermal transport properties in the corresponding sublayer.^[^ ^3^ ^]^ In recent years, superconductivity was discovered in a series of BiS_2_‐based layered compounds consisted of BiS_2_ superconducting layers and insulating layers like Bi_2_O_2_ or La_2_O_2_, which have been carefully studied because of their structural similarity to high‐temperature cuprate and iron‐based superconductors.^[^ ^4^ ^]^

The first BiS_2_‐based layered superconductor Bi_4_O_4_S_3_ composed of BiS_2_ superconducting layers, Bi_2_O_2_ and SO_4_ blocking layers shows superconductivity at 4.5 K that was reported in 2012.^[^ ^4a^ ^]^ By replacing the blocking layer as in other superconducting families, a number of BiS_2_‐based layered superconductors have been found with various cationic blocking layers, such as RE_2_O_2_ (RE = La, Ce, Pr, Nd, Sm, and Yb),^[^ ^4^, ^5^ ^]^ AE_2_F_2_ (AE = Sr, Eu),^[^ ^6^ ^]^ and Eu_3_F_4_^[^ ^7^ ^]^ layers in addition to Bi_2_O_2_ layer. And the SO_4_ layer in Bi_4_O_4_S_3_ can also be replaced by S─S dimer.^[^ ^8^ ^]^ However, the flexibility of the superconducting layer is very limited. There are only three types of superconducting layers to date, BiS_2_ layer including S partially or completely substituted by Se,^[^ ^9^ ^]^ BiS_2_Cl layer,^[^ ^10^ ^]^ and *M*_4_S_6_ (*M*_4_ = Bi_2_Pb_2_, Bi_3_Ag or Bi_3_Ag_0.6_Sn_0.4_, etc) layer.^[^ ^11^ ^]^ In these BiS_2_‐based compounds, bismuth binds five sulfur atoms forming a pyramid configuration in BiS_2_ layer and *M*_4_S_6_ layer,^[^ ^4^, ^5^, ^6^, ^7^, ^8^, ^11^ ^]^ whereas bismuth is surrounded by two sulfur atoms and four chlorine atoms forming a six‐coordinated configuration in BiS_2_Cl layer.^[^ ^10^ ^]^

For cuprate superconductors, CuO_2_ layer is dominant for the superconductivity, in which Cu‐O has the tetracoordinated square‐planar configuration (Nd_2‐x_Ce_x_CuO_4_),^[^ ^12^ ^]^ pentacoordinated pyramid configuration (Bi‐2212 and (Nd_1‐x‐y_Ce_x_Sr_y_)_2_CuO_4_),^[^ ^2^, ^13^ ^]^ or hexacoordinated octahedron configuration ((La,Sr)_2_CuO_4_ and Tl_2_Ba_2_CuO_6_).^[^ ^14^ ^]^ These variously coordinated copper in CuO_2_ layer greatly enrich the crystal structures of cuprate superconductors. Meanwhile, cuprates having CuO_2_ layers with differently coordinated copper show varying *T*_c_s and carrier types.^[^ ^2^, ^12^, ^13^, ^14^, ^15^ ^]^ Especially, HgBa_2_Ca_2_Cu_3_O_8+x_ owning both the tetracoordinated (square‐planar configuration) and pentacoordinated (pyramid configuration) copper keeps the highest *T*_c_ ≈133 K for all superconductors under ambient pressure.^[^ ^16^ ^]^ Compared to copper, bismuth can bind to different ligands and form various complexes with coordination number up to 10 owing to its stronger deformability and polarizability.^[^ ^17^ ^]^ However, there are only three types of superconducting layer and two coordination structures of bismuth in BiS_2_‐based superconductors. We noticed that bismuth can be octahedrally coordinated by six sulfur atoms to form a BiS_6_ cluster in ABiS_2_ (A = alkali metal) and TlBiS_2_,^[^ ^18^ ^]^ which hints that the six‐coordinated bismuth‐based complex is a potential structural unit forming new BiS‐based layered materials.

In this work, we synthesized a new superconductor Bi_5_O_4_S_3_Cl containing BiS_3_ superconducting layers with six‐coordinated bismuth, which is distinct from all the known BiS‐based superconducting materials. Bulk superconductivity was observed with a *T_c_* above 3 K. The first‐principles calculations indicate that sulfur vacancies introducing carriers may induce the superconductivity. The discovery of this new BiS_3_ superconducting layer sheds light on the exploration for more BiS‐based superconductors.

## 2 Results and Discussion

**Figure 1:**
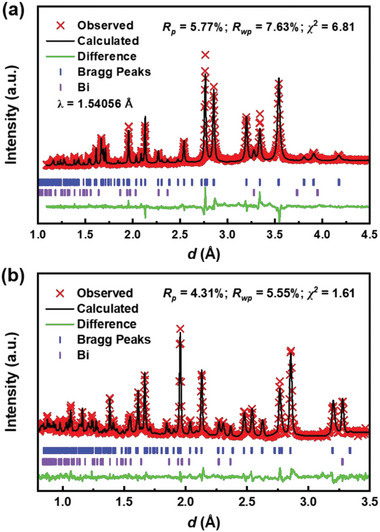
a) The PXRD pattern and b) NPD pattern with the results of refinement for polycrystalline Bi_5_O_4_S_3_Cl.

**Figure** **1**a shows the powder X‐ray diffraction pattern (PXRD) and the Rietveld refinement results for polycrystalline Bi_5_O_4_S_3_Cl. The pattern can be indexed by a tetragonal structure with *P*4/*mmm* (No.123) space group having side phase Bi (2.84%). The lattice parameters are *a* = 3.9104(9) Å and *c* = 16.7064(04) Å, which are smaller than those of Bi_3_O_2_S_2_Cl.^[^ ^10^ ^]^ To further distinguish the atomic position of sulfur and chlorine, we conducted neutron powder diffraction (NPD) as sulfur and chlorine have neutron coherent scattering lengths of 2.85 fm and 9.58 fm, respectively. As shown in Figure 1b, the NPD pattern shows that Bi_5_O_4_S_3_Cl has a *P*4/*mmm* space group with lattice parameters *a* = 3.9148(4) Å and *c* = 16.7220(9) Å, which are in agreement with those of PXRD. The profile residual (*R*_p_), weighted profile residual (*R*_wp_), and goodness of fit (*χ* ^2^) are reasonable and demonstrate the reliability of the refinements. The obtained crystallographic data and atomic coordinates for PXRD and NPD data are listed in Tables S1 and S2 (Supporting Information). It is noticeable that there are defects at S1 sites and the occupancy of S1 sites is ≈0.97 both for PXRD and NPD data. The existence of sulfur vacancies at S1 sites has been demonstrated in BiS_2_Cl layer of Bi_3_O_2_S_2_Cl.^[^ ^10^ ^]^

**Figure 2:**
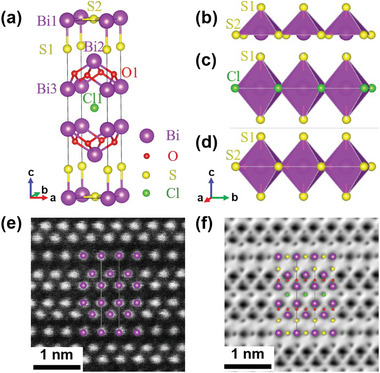
a) The side view of crystal structure of Bi_5_O_4_S_3_Cl. b) The schematic of pentacoordinated pyramid configuration of bismuth in the BiS_2_ layer, and the hexacoordinated octahedron configuration of bismuth in the c) BiS_2_Cl layer and d) BiS_3_ layer. The atomically resolved e) HAADF and f) ABF images of Bi_5_O_4_S_3_Cl single crystal along the [100] projection.

A schematic of Bi_5_O_4_S_3_Cl crystal structure is shown in **Figure** **2**a, which is consisted of BiS_3_ layers and Bi_2_O_2_ layers alternately stacking along the *c*‐axis with a Cl atom located at the center. In the unique BiS_3_ layer, six sulfur atoms form an octahedron and a bismuth atom occupies the center of the octahedron shown in Figure 2d, which is different from the BiS_2_ layer and the BiS_2_Cl layer as shown in Figure 2b,c. The typical bond lengths and bond angles of Bi_5_O_4_S_3_Cl (NPD), Bi_3_O_2_S_2_Cl, Bi_2_OS_2_, Bi_4_O_4_S_3_, and LaOBiS_2_ are listed in Table S3 (Supporting Information).^[^ ^10^, ^19^ ^]^ The bond length of Bi2‐O1 and bond angle of O1‐Bi2‐O1 of Bi_5_O_4_S_3_Cl are 2.410(5) Å and 108.6(3)° respectively, which are similar to those of other compounds.^[^ ^10^, ^19^ ^]^ For Bi_5_O_4_S_3_Cl, the corresponding bond lengths of Bi1‐S1 and Bi1‐S2 are 2.732(1) Å and 2.768(2) Å, which are close to those of Bi_3_O_2_S_2_Cl.^[^ ^10^ ^]^ Compared with BiS_2_‐based layered compounds with pentacoordinated bismuth in Table S3 (Supporting Information), the bond length of Bi1‐S1 in Bi_5_O_4_S_3_Cl is much longer than that in Bi_2_OS_2_, Bi_4_O_4_S_3_, and LaOBiS_2_, whereas the bond length of Bi1‐S2 is a little bit shorter.^[^ ^19^ ^]^ Meanwhile, there should be no distortion in the BiS_3_ layer as the bond angle of S1‐Bi1‐S2 is 90° in Bi_5_O_4_S_3_Cl.

A typical optical image of Bi_5_O_4_S_3_Cl single crystal is shown in Figure S1 (Supporting Information). The scanning transmission electron microscopy (STEM) high‐angle annular dark‐field (HAADF) image and annular bright‐field (ABF) image of Bi_5_O_4_S_3_Cl single crystal along the [100] axis using high‐resolution aberration‐corrected STEM are shown in Figure 2e,f, respectively. In accordance with the relationship between scattering intensity and *Z* (*Z* is the atomic number of an element), the bright dots denote bismuth atoms in the HAADF image. In the ABF image, all the lighter atoms such as oxygen, sulfur, and chlorine, are clearly observed at the sites between bismuth atoms. Hence, the HAADF and ABF images further confirm the unique BiS_3_ layer and Cl layer in Bi_5_O_4_S_3_Cl and its crystal structure is distinct from that of Bi_3_O_2_S_2_Cl.^[^ ^10^ ^]^

**Figure 3:**
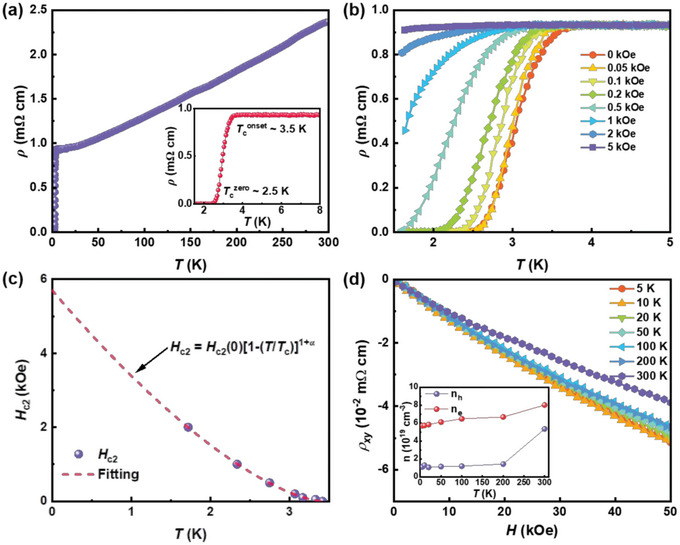
a) The temperature‐dependent resistivity of polycrystalline Bi_5_O_4_S_3_Cl. b) The resistivity of polycrystalline Bi_5_O_4_S_3_Cl at low temperature under various magnetic fields. c) The critical field as a function of temperature. The dotted line shows fitting by the equation *H_c2_* (*T*) = *H*_c2_(0) (*1 –* (*T*/*T*_c_))^1+^ *^α^*. d) The Hall resistivity *ρ_xy_* versus magnetic field at 5 K, 10 K, 20 K, 50 K, 100 K, 200 K, and 300 K. The inset shows the derived carrier concentration.

The temperature‐dependent resistivity of polycrystalline Bi_5_O_4_S_3_Cl is shown in **Figure** **3**a. Below 300 K, the resistivity monotonously decreases with decreasing temperature, which is similar to that of Bi_4_O_4_S_3_, Bi_3_O_2_S_2_Cl, Bi_2_(O, F)S_2_, Bi_3_O_2_S_3_, and LaO_1‐x_F_x_BiSSe.^[^ ^4^, ^10^, ^20^ ^]^ As the temperature is down to 3.5 K, a sharp drop in resistivity is observed, indicating a superconducting transition (*T*_c_^onset^ ≈3.5 K, d*ρ*/d*T* >0). Zero resistance is observed at *T*_c_^zero^ ≈2.5 K. As shown in Figure 3b, the drop of resistivity is suppressed and the superconducting transition broadens with increasing magnetic field. *T_c_* is determined by 90% *ρ_n_* (the normal resistivity upon *T_c_* ^onset^) here. The temperature‐dependent upper critical field (*H*_c2_) is plotted in Figure 3c. *H*_c2_ of Bi_5_O_4_S_3_Cl has an upward curvature close to *T*_c_ (*H* = 0), which has also been observed in BiS_2_‐based superconductors like Bi_4_O_4_S_3_, La_1‐x_ *M*_x_OBiS_2_ (*M* = Th, Hf), and EuFBiS_2._^[^ ^6^, ^21^ ^]^ This upward curvature of *H*_c2_ is considered to be a signature of multi‐band superconductivity in MgB_2_ and *T*_d_‐MoTe_2_,^[^ ^22^ ^]^ implying that an unconventional pairing mechanism possibly exists in BiS‐based superconductors. Deviating from the Werthamer–Helfand–Hohenberg theory based on the single‐band model,^[^ ^23^ ^]^ the *H*_c2_ curve of Bi_5_O_4_S_3_Cl can be well fitted by using the empirical expression *H_c2_* (*T*) = *H*_c2_(0) (*1 –* (*T*/*T*_c_))^1+^ *^α^*,^[^ ^24^ ^]^ where *H*_c2_(0) and *α* are fitting parameters. The estimated *H*_c2_(0) is ≈5.8 kOe, which yields a Ginzburg‐Landau length *ξ*_GL(0)_ [ = $\sqrt{\Phi_{0}/2\pi \mu_{0}H_{c2}\left(0\right)}$] of ≈23.8 nm.

To clarify the carrier's type as well as its concentration in the sample, we measured the magnetic‐field‐dependent Hall resistivity (*ρ_xy_*) at different temperatures. As shown in Figure 3d, *ρ_xy_* with negative slope at all measured temperatures indicates that electron‐like carriers are dominant. With the increase of magnetic field, *ρ_xy_* exhibits a slight deviation from the linear behavior, indicating its multi‐band electronic structure and being consistent with the calculated band structure. This nonlinear characteristic of *ρ_xy_* is also observed in Bi_3_O_2_S_2_, Bi_4_O_4_S_3_, Eu_3_F_4_Bi_2_S_4_, and CeO_1‐x_F_x_BiS_2_,^[^ ^5^, ^20^, ^25^ ^]^ but it's different from the linear behavior in Bi_3_O_2_S_2_Cl and REO_1‐x_F_x_BiS_2_ (RE = La, Pr, and Nd).^[^ ^10^, ^26^ ^]^ Based on the results of band structure (as discussed later), we determined the carrier density *n* with a simple two‐band model.^[^ ^27^ ^]^

(1) $$\;\rho_{\mathrm{xy}}=\;-\frac{\;H}{e}\frac{\left(\mu_{e}^{2}n_{e}-\mu_{h}^{2}n_{h}\right)+{\left(\mu_{e}\mu_{h}H\right)}^{2}\left(n_{e}-n_{h}\right)}{{\left(\mu_{e}n_{e}+\mu_{h}n_{h}\right)}^{2}+{\left(\mu_{e}\mu_{h}H\right)}^{2}{\left(n_{e}-n_{h}\right)}^{2}}$$

(2) $$\rho_{xx}\;\left(0\right)=\frac{1}{e\left(\mu_{e}n_{e}+\mu_{h}n_{h}\right)}$$

Here, *n_e_* (or *n_h_*) is the carrier density of electrons (or holes), *µ*_e_ (*µ*_h_) the mobility of electrons (holes), and *ρ_xx_* (0) the longitudinal resistivity at 0 T. As shown in the inset of Figure 3d, the electron‐like carrier density *n_e_* of Bi_5_O_4_S_3_Cl is ≈5.7 × 10^19^ cm^−3^ at 5 K, which is comparable with those of Bi_4_O_4_S_3_ (1.53 × 10^19^ cm^−3^ @ 10 K), CeO_1‐x_F_x_BiS_2_ (≈10^19^ cm^−3^ @ 2 K), Sr_0.5_La_0.5_FBiS_2_ (1.05 × 10^19^ cm^−3^ @ 5 K), Bi_3_O_2_S_3_ (1.45 × 10^19^ cm^−3^ @ 2 K), and Bi_3_O_2_S_2_Cl (9 × 10^19^ cm^−3^ @ 5 K).^[^ ^5^, ^6^, ^10^, ^20^ ^]^ Such a low carrier density seems to be a common feature for BiS‐based superconductors. The theoretical calculation reveals a good nesting condition in Bi_4_O_4_S_3_ and LaO_0.5_F_0.5_BiS_2_.^[^ ^28^ ^]^ Moreover, it was reported a charge density wave occurs in NdO_0.7_F_0.3_BiS_2_.^[^ ^29^ ^]^ The strong electron correlation in BiS‐based materials may be one of the main reasons for the low‐carrier‐density superconductivity.

**Figure 4:**
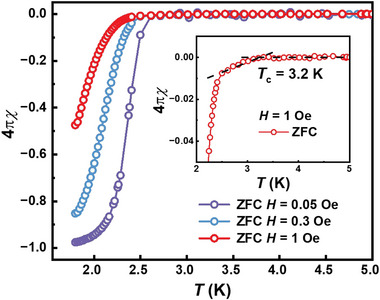
The ZFC magnetic susceptibility of polycrystalline Bi_5_O_4_S_3_Cl corrected for the demagnetization factor under magnetic fields of 0.05 Oe, 0.3 Oe, and 1 Oe. The inset displays the enlarged magnetic susceptibility under a magnetic field of 1 Oe.

**Figure** **4** shows the temperature‐dependent magnetic susceptibility, corrected for the demagnetization factor, under small magnetic fields of 0.05 Oe, 0.3 Oe, and 1 Oe with zero‐filed cooling (ZFC) mode for polycrystalline Bi_5_O_4_S_3_Cl. Under a magnetic field of 0.05 Oe, the superconducting shielding fraction is estimated to be 97% at 1.8 K, indicating the bulk superconductivity in Bi_5_O_4_S_3_Cl. The inset in Figure 4 displays the enlarged magnetic susceptibility under the magnetic field of 1 Oe. *T_c_*, determined by the intersection of the extrapolated normal susceptibility to lower temperatures and the slope of the diamagnetic signal in the ZFC data,^[^ ^30^ ^]^ is 3.2 K. In addition, the temperature‐dependent magnetic susceptibility of Bi_5_O_4_S_3_Cl single crystals under a magnetic field of 10 Oe is shown in Figure S2 (Supporting Information), in which a clear superconducting transition is also observed at 3.2 K. Figure S3 (Supporting Information) displays the magnetization data with applied field over a range of temperature below *T*_c_, which suggests that Bi_5_O_4_S_3_Cl is a type‐II superconductor. The lower critical field is ≈20 Oe at 1.8 K defined as the field at which *M* – *H* curve starts to deviate from the Meissner effect. It's a medium value compared with that of BiS_2_‐based superconductors, like PrO_0.5_F_0.5_BiS_2_ (8 Oe at 2.1 K), Bi_4_O_4_S_3_ (15 Oe at 2 K) and EuSr_2_Bi_2_S_2_Se_2_F_4_ (≈100 Oe under 0.86 GPa at 2 K).^[^ ^5^, ^20^, ^31^ ^]^

Note that S1 sites of Bi_5_O_4_S_3_Cl are deficient based on PXRD and NPD data. The existence of sulfur vacancies is also confirmed by energy‐dispersive spectroscopy (EDS) spectra shown in Figure S4 (Supporting Information) both in polycrystalline and single crystal Bi_5_O_4_S_3_Cl. As the synthesis temperature and decomposition temperature of Bi_5_O_4_S_3_Cl are very close (Figure S5, Supporting Information), sulfur vacancies are probably formed during the synthesizing process. Single crystals with relatively higher growing temperature having higher concentration of sulfur vacancies further prove it. We speculate that the superconductivity of Bi_5_O_4_S_3_Cl is correlated with the sulfur vacancies, which is also reported to be the origin of superconductivity in Bi_3_O_2_S_2_Cl.^[^ ^10^ ^]^ In fact, for BiS_2_‐based superconductors, the parent compounds are insulators or semiconductors, whereas superconductivity can only emerge with carrier doping by inducing vacancies or non‐equivalent element substitution.^[^ ^4^, ^10^, ^20^ ^]^

To further understand the effect of sulfur deficiency on band structure, we calculated the electronic band structures, partial density of states (PDOS), and total density of states (TDOS) of Bi_5_O_4_S_x_Cl (*x* = 3, 2.9, 2.857, 2.8, 2.75, and 2.67) using first‐principles calculations. The first Brillouin zone of Bi_5_O_4_S_3_Cl and the calculation path of band structure are shown in Figure S6a (Supporting Information). Tables S4 and S5 (Supporting Information) show the corresponding supercells and optimized structural parameters for each phase. As shown in Figures S7 and S9 (Supporting Information), the band structure evolution can be clearly seen with the increasing sulfur vacancies at S1 site and S2 site. It is found that Bi_5_O_4_S_x_Cl (*x* = 2.9, 2.857, 2.8, 2.75, and 2.67) is metallic, in contrast to the semiconducting feature of the parent phase Bi_5_O_4_S_3_Cl. Moreover, we determined the relative energy difference between vacancies at S1 site and S2 site with the same concentration (Table S6, Supporting Information), which indicates the sulfur vacancy prefers S1 site and can extend to S2 site with increasing vacancy concentration. The TDOS of Bi_5_O_4_S_x_Cl with vacancies located at S1 site (S1‐vacancies, Figure S8, Supporting Information) increases much near the Fermi level over the case with vacancies located at S2 site (S2‐vacancies, Figure S10, Supporting Information). The enhanced TDOS at the Fermi level may play an important role in promoting the superconductivity.

**Figure 5:**
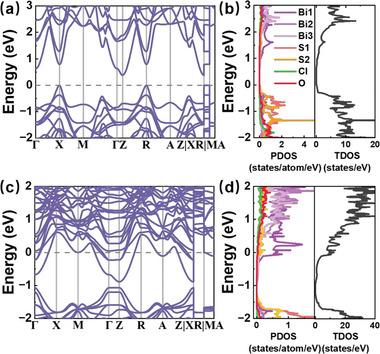
The a) band structure and b) PDOS (left panel), TDOS (right panel) of Bi_5_O_4_S_3_Cl. The c) band structure and d) PDOS (left panel), TDOS (right panel)) of Bi_5_O_4_S_2.75_Cl (S1‐vacancies, all the sulfur vacancies at the S1 site).

For a clear comparison, the band structures and PDOS (TDOS) of Bi_5_O_4_S_3_Cl and Bi_5_O_4_S_2.75_Cl (S1‐vacancies) are shown in **Figure** **5**. The valance band of Bi_5_O_4_S_3_Cl from – 2 to 0 eV shown in Figure 5b is mainly contributed by the *p* electrons of S1 and S2 atoms. Besides, the fat band (Figure S6b, Supporting Information) of Bi_5_O_4_S_3_Cl shows the hybridization between bismuth and sulfur atoms (S2) on the conduction band, which is consistent with that of LaOBiS_2_.^[^ ^32^ ^]^ As depicted in Figure 5c, the topology of Fermi surface of Bi_5_O_4_S_2.75_Cl changes a lot and the Fermi level crosses both electron pockets and hole pockets in comparison with that of Bi_5_O_4_S_3_Cl. The band structure of Bi_5_O_4_S_2.75_Cl differs from that of Sr_1‐x_La_x_FBiS_2_ and REO_1‐x_F_x_BiS_2_ (RE = La, Ce, Pr, and Nd), in which the element doping doesn't change the crystal structure and just lifts the Fermi level leaving only electron pockets near the Fermi level.^[^ ^32^, ^33^ ^]^ Furthermore, the TDOS (Figure 5d) of Bi_5_O_4_S_2.75_Cl at Fermi level increases much. In particular, the PDOS shows that Fermi level is on the peak position of the Bi1 6*p* orbitals and S2 3*p* orbitals, suggesting that the plane square lattice (Bi1‐S2) is dominant in the active superconducting BiS_3_ layer. This feature is similar to that of BiS_2_‐based layered superconductors, in which the local density of states show that charges are localized in Bi‐S plane, whereas the S outside of BiS_2_ layer just gathers a few electrons.^[^ ^28^, ^33^ ^]^ Thus, it can be inferred that layered superconductors with BiS_3_ layer or BiS_2_ layer probably have a similar superconducting mechanism.

## 3 Conclusion

In summary, we successfully synthesized a new layered compound Bi_5_O_4_S_3_Cl containing Bi_2_O_2_ layers and unique BiS_3_ layers stacking along the *c*‐axis and having a Cl atom located at the center. The resistivity and magnetic susceptibility measurements show a clear superconducting transition above 3 K. Carrier doping introduced by sulfur vacancies is deduced to result in the superconductivity. And further studies will be needed to understand the origin of superconductivity in Bi_5_O_4_S_3_Cl. This new superconductor with unique BiS_3_ layer sheds light on the discovery of new BiS‐based superconducting materials.

## 4 Experimental Section

### Polycrystalline Sample Preparation

Polycrystalline Bi_5_O_4_S_3_Cl was synthesized via solid‐state reaction using high‐purity starting materials Bi_2_O_3_ (Innochem, 99.999%), Bi_2_S_3_ (Innochem, 99.999%), and BiOCl (Alfa Aesar, 99.999%). Starting materials were mixed at a certain ratio and ground, pressed into a pellet, and then put into an alumina crucible, which was sealed in a quartz tube. After that, the sample was slowly heated to 773 K, held for 24 h, and then cooled down to room temperature over 20 h. The product was then ground, pressed into a pellet, and sintered under vacuum at 773 K for another 24 h, followed by cooling down to room temperature over 20 h. To figure out the origin of superconductivity in Bi_5_O_4_S_3_Cl, the carriers of polycrystalline Bi_5_O_4_S_3_Cl are modulated by introducing chlorine vacancies or sulfur vacancies but failed. Further studies will be needed to understand the origin of superconductivity in Bi_5_O_4_S_3_Cl.

### Single Crystal Preparation

Great efforts have been made in single crystal growth, such as flux method using Bi/Pb/In/LiCl/KCl/CsCl/PbCl_2_/AgCl as flux and vapor transport method using I_2_/BiOCl/BiCl_3_ as transport agent. Unfortunately, all these attempts failed. A small number of Bi_5_O_4_S_3_Cl single crystals can be obtained occasionally on the surface of pellet sintered at 778 K under vacuum for 120 h in a few bathes. The typical size of crystals is ≈10 µm – 30 µm as shown in Figure S1 (Supporting Information). These single crystals accompanied by some polycrystalline samples were collected and measured the temperature‐dependent magnetic susceptibility shown in Figure S2 (Supporting Information). Once the sintering temperature is above 803 K, bismuth and other phases will form on the surface of pellet due to the decomposition of polycrystalline Bi_5_O_4_S_3_Cl.

### Structure Characterization

PXRD data were collected on a PANalytical X'Pert PRO diffractometer (Cu K*α* radiation) operated at 40 kV voltage and 40 mA current with a graphite monochromator in a reflection mode (2*θ* = 5 – 90°, step 0.017°). NPD was conducted on the general‐purpose powder diffractometer with time‐of‐fight technique at the China spallation neutron source. Indexing and Rietveld refinement were performed using the DICVOL06 and FULLPROF programs.^[^ ^34^ ^]^ The schematic crystal structure was depicted using VESTA.^[^ ^35^ ^]^ The elemental analysis was carried out using a scanning electron microscope (SEM, Hitachi S‐4800) equipped with an electron microprobe analyzer for the semiquantitative elemental analysis in the EDS mode. STEM specimens of a single crystal with suitable thickness were prepared by focused ion beam on Helios Nanolab 600i. The atomic structure of samples was characterized using a JEM‐ARM 200CF S/TEM operated at 200 kV with the STEM HAADF imaging and ABF imaging.

### Physical Properties Measurements

The superconducting signal was measured on MPMS‐XL1 (Quantum Design) with a tiny remnant field < 4 mOe to avoid the interference on superconducting signal from remnant field. The ZFC magnetic susceptibility of polycrystalline Bi_5_O_4_S_3_Cl was measured under small magnetic fields of 0.05 Oe, 0.3 Oe, and 1 Oe. Temperature‐dependent electrical resistivity was measured on a physical property measurement system (Quantum Design), whereas field‐dependent magnetization measurements were carried out on a vibrating sample magnetometer (Quantum Design). Contacts for standard four‐probe configuration were made by attaching platinum wires using silver paint. The dimensions of polycrystalline Bi_5_O_4_S_3_Cl sample were 2.5 mm in length, 2.3 mm in width, and 0.6 mm in thickness.

### Thermogravimetric Analysis (TGA)

The TGA of polycrystalline Bi_5_O_4_S_3_Cl is performed on SDT Q600 (TA Instruments) at a rate of 5 C° min^−1^ in an argon atmosphere.

### First‐Principles Calculations

The first principles calculations were carried out with density functional theory (DFT) implemented in the Vienna ab initio simulation package (VASP).^[^ ^36^ ^]^ The generalized gradient approximation (GGA) was adopted in the form of the Perdew‐Burke‐Ernzerhof (PBE) for the exchange correlation potentials.^[^ ^37^ ^]^ The projector‐augmented‐wave (PAW)^[^ ^38^ ^]^ pseudopotentials were used with a plane wave energy of 600 eV. A Monkhorst‐Pack Brillouin zone sampling grid^[^ ^39^ ^]^ with a resolution of 0.02 × 2π Å^−1^ was applied. Atomic positions and lattice parameters were relaxed until all the forces on the ions were less than 10^−2^ eV Å^−1^ and the energy difference between two adjacent ionic steps was less than 5 × 10^−6^ eV per atom. For Bi_5_O_4_S_x_Cl (*x* = 2.9, 2.857, 2.8, 2.75, and 2.67) with sulfur vacancies, corresponding super cell was constructed with certain sulfur atom removed from the supercell. The corresponding supercell and optimized structural parameters are shown in Tables S4 and S5 (Supporting Information). For the unfolding of supercell energy bands, the VASPKIT package^[^ ^40^ ^]^ was used to calculate the effective bands.

CCDC‐2241408 contains the supplementary crystallographic data for this paper. These data can be obtained free of charge from the Cambridge Crystallographic Data Centre via www.ccdc.cam.ac.uk/data_request/cif.

## Conflict of Interest

The authors declare no conflict of interest.

## Supporting information

Supporting Information

## Data Availability

The data that support the findings of this study are available from the corresponding author upon reasonable request.
