# Supplementary material for: Discovery of a Superconductor Bi5O4S3Cl Containing the Unique BiS3 Layer
Source: Adv Sci (Weinh). 2023 Aug 27;10(30):2303569. doi: 10.1002/advs.202303569 (PMC10602514; doi:10.1002/advs.202303569)
Supplement: Supplementary file 1 — Supporting Information [file ADVS-10-2303569-s001.pdf]

ADVANCED SCIENCE
Open Access

Supporting Information

for *Adv. Sci.*, DOI 10.1002/advs.202303569

Discovery of a Superconductor $Bi_5O_4S_3Cl$ Containing the Unique BiS_3 Layer

Yaling Yang, Xiao Fan, Jiali Liu, Cheng Cao, Zhaolong Liu, Jun Deng, Ting Lin, Qinghua Zhang, Ke Liao, Xiaoli Dong, Gang Wang and Xiaolong Chen**

Supporting Information

Discovery of a Superconductor $Bi_5O_4S_3Cl$ Containing the Unique BiS_3 Layer

Yaling Yang, Xiao Fan, Jiali Liu, Cheng Cao, Zhaolong Liu, Jun Deng, Ting Lin, Qinghua Zhang, Ke Liao, Xiaoli Dong, Gang Wang, and Xiaolong Chen**

1. Supplementary figures

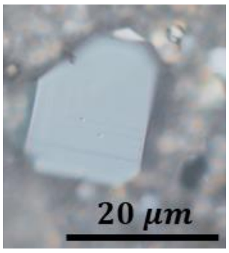

Figure S1. An optical image of $Bi_5O_4S_3Cl$ single crystal. The typical size of $Bi_5O_4S_3Cl$ single crystals is about 10 um - 30 um.

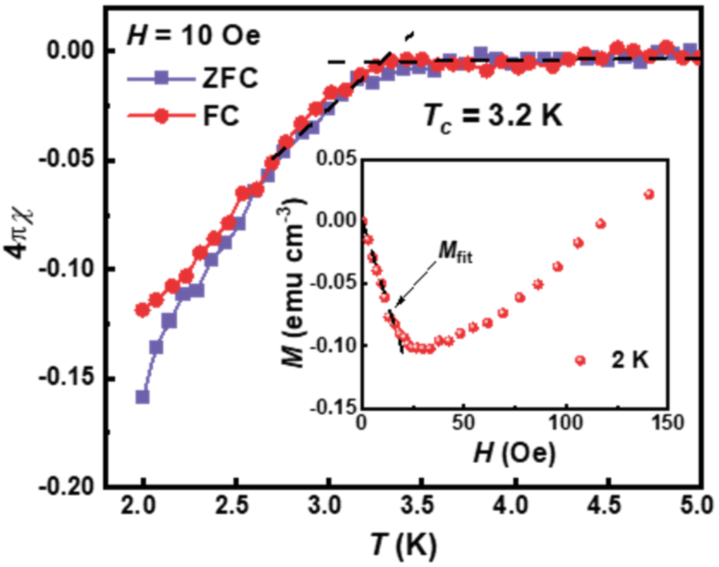

Figure S2. The temperature-dependent magnetic susceptibility of $Bi_5O_4S_3Cl$ single crystals. The inset displays the isothermal magnetization with magnetic field at 2 K. The deviation from linear fitting marks the lower critical field as ~18 Oe at 2 K.

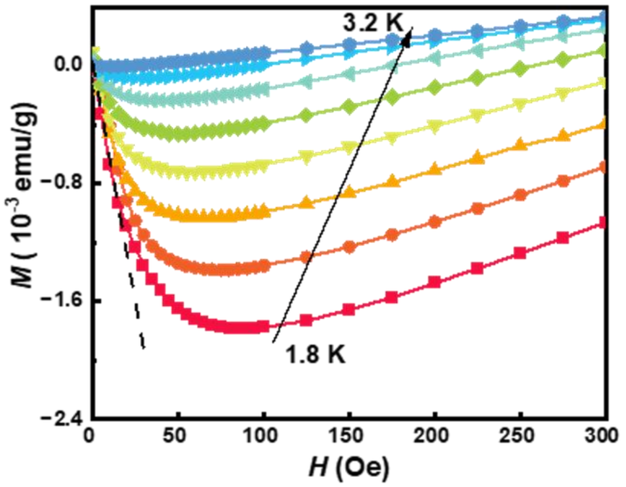

Figure S3. Magnetization data with applied field of polycrystalline $Bi_5O_4S_3Cl$ at various temperatures below transition temperature. The lower critical field is about 20 Oe determined by the point of magnetization deviating from Meissner effect at 1.8 K.

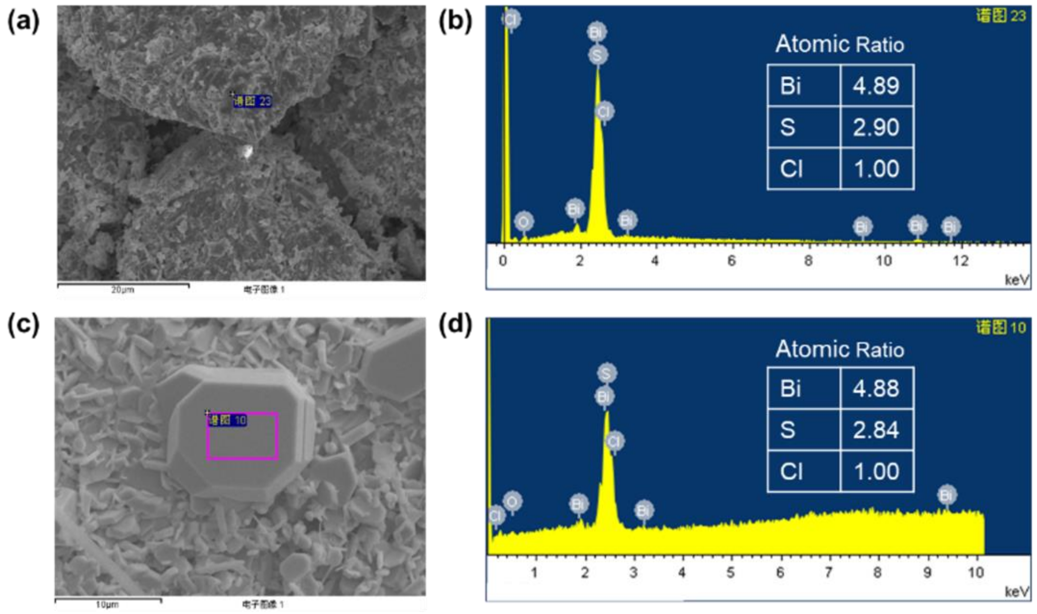

Figure S4. The scanning transmission electron microscopy images and the energy-dispersive spectroscopy spectrum of (a), (b) polycrystalline $Bi_5O_4S_3Cl$ and (c), (d) $Bi_5O_4S_3Cl$ single crystal, respectively. The obtained atomic ratios are shown in the insets of Figure S4b and Figure S4d.

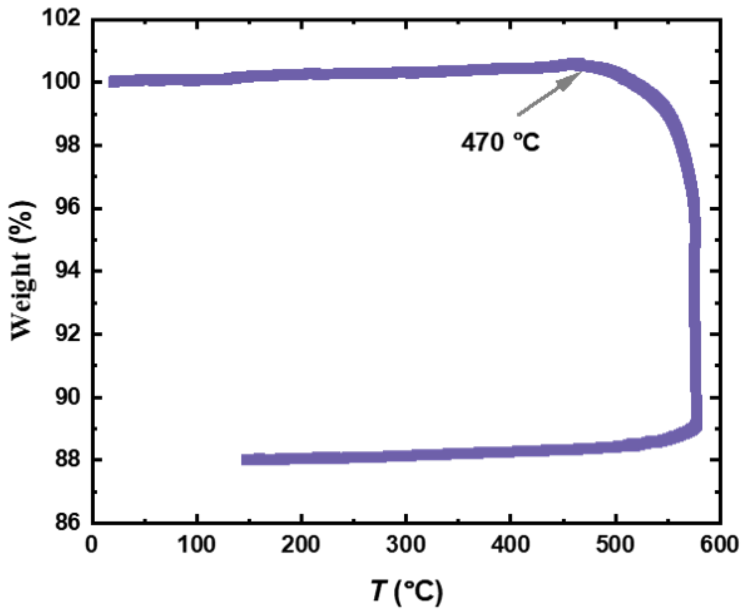

Figure S5. The thermogravimetric analysis curve of polycrystalline $Bi_5O_4S_3Cl$.

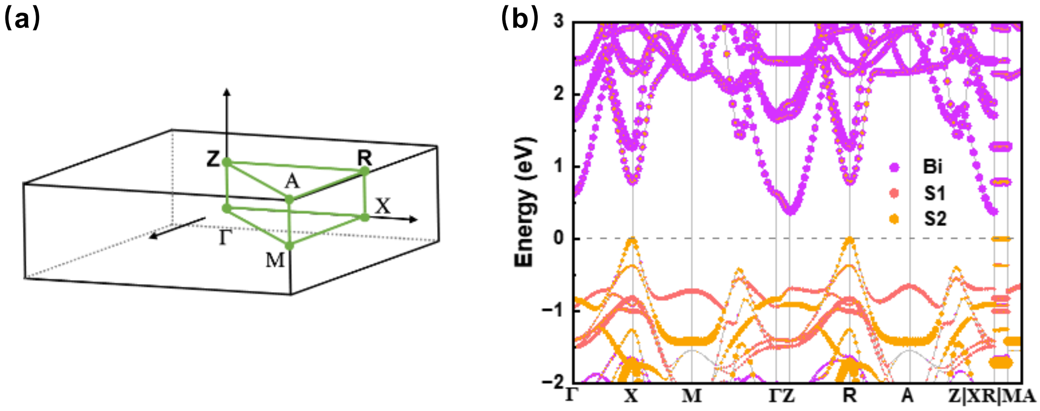

Figure S6. (a) The first Brillouin zone of $Bi_5O_4S_3Cl$ and the calculation path of band structure. (b) The fat band of $Bi_5O_4S_3Cl$.

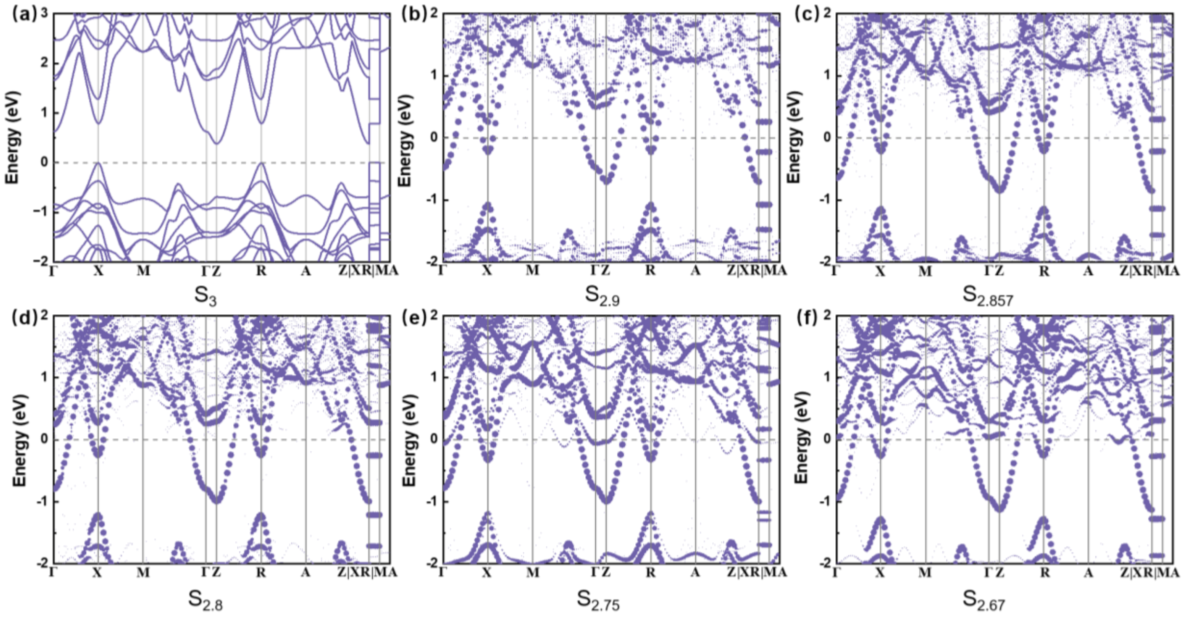

Figure S7. The unfolded band structures of $Bi_5O_4S_xCl$ with x = 3, 2.9, 2.857, 2.8, 2.75, and 2.67. The sulfur vacancy is located at S1 site.

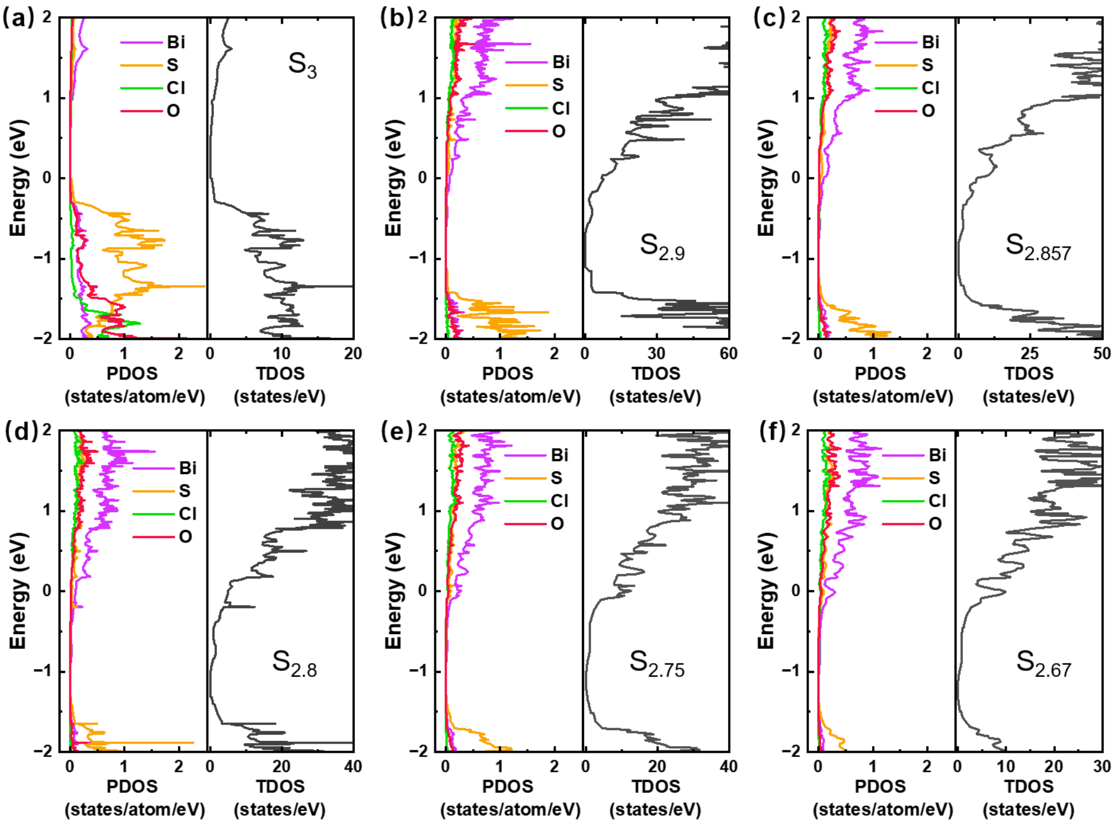

Figure S8. The partial density of states (PDOS, left panel) and total density of states (TDOS, right panel) of $Bi_5O_4S_xCl$ with x = 3, 2.9, 2.857, 2.8, 2.75, and 2.67. The sulfur vacancy is located at S1 site.

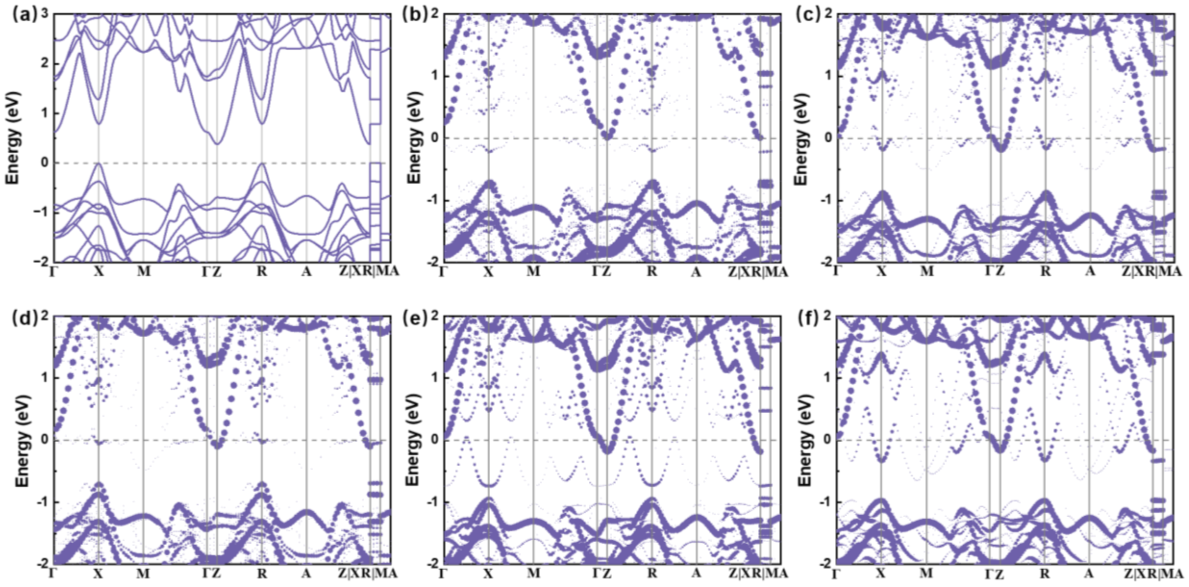

Figure S9. The unfolded band structures of $Bi_5O_4S_xCl$ with x = 3, 2.9, 2.857, 2.8, 2.75, and 2.67. The sulfur vacancy is located at S2 site.

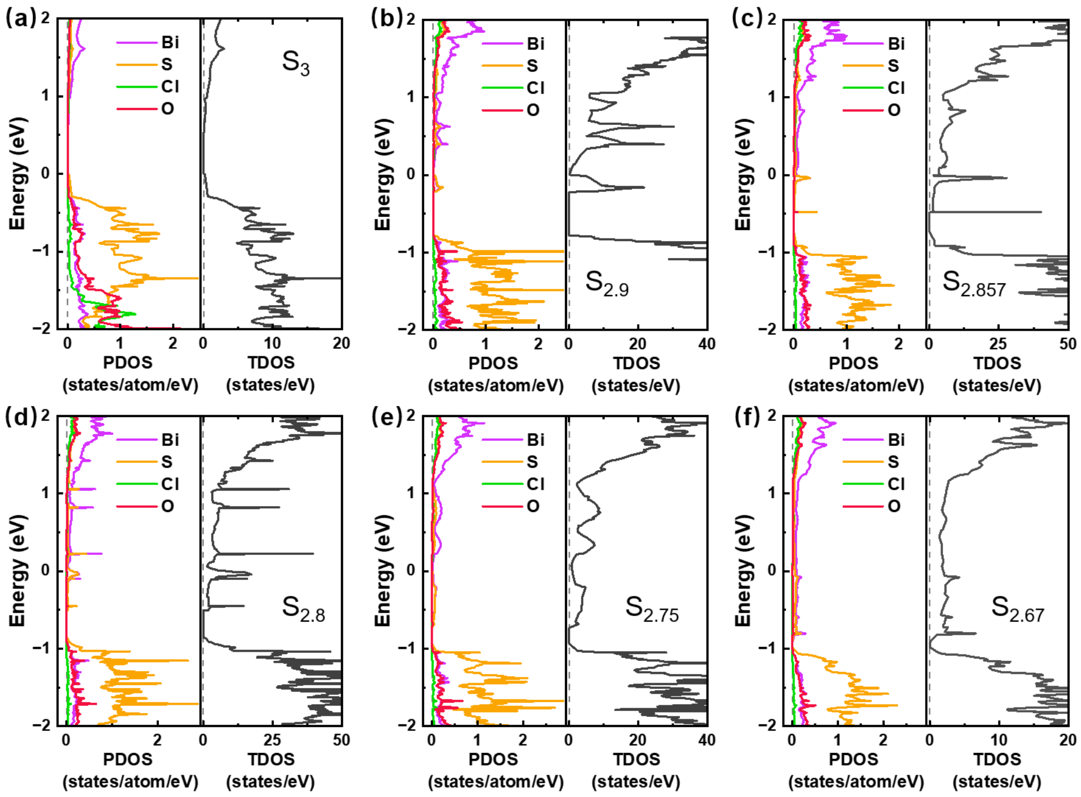

Figure S10. The PDOS (left panel) and TDOS (right panel) of $Bi_5O_4S_xCl$ with x = 3, 2.9, 2.857, 2.8, 2.75, and 2.67. The sulfur vacancy is located at S2 site.

2. Supplementary tables

Table S1. The crystallographic data and atomic coordinates obtained from Rietveld refinement against powder X-ray diffraction data for polycrystalline $Bi_5O_4S_3Cl$.

	Parameter			Value		
	a (Å)			3.9104(9)		
	c (Å)			16.7064 (04)		
	V (Å^3)			255.47(3)		
Atom	*Wyck.*	*x*/*a*	*y*/*b*	*z*/*c*	Occupancy	*U* (Å^2)
Bi1	1*a*	1	1	1	1	0.022
Bi2	2*h*	0.5	0.5	0.7654(3)	1	0.013
Bi3	2*g*	1	1	0.6121	1	0.001
S1	2*g*	1	1	0.8329(9)	0.97(3)	0.014
S2	1*c*	0.5	0.5	1	1	0.060
Cl1	1*d*	0.5	0.5	0.5	1	0.062
O1	4*i*	1	0.5	0.6916(1)	1	0.057

Table S2. The crystallographic data and atomic coordinates obtained from Rietveld refinement against neutron powder diffraction (NPD) data for polycrystalline $Bi_5O_4S_3Cl$.

	Parameter			Value		
	a (Å)			3.9148 (4)		
	c (Å)			16.7220(9)		
	V (Å^3)			256.28(1)		
Atom	*Wyck.*	*x*/*a*	*y*/*b*	*z*/*c*	Occupancy	*U* (Å^2)
Bi1	1*a*	1	1	1	1	0.084
Bi2	2*h*	0.5	0.5	0.7643(02)	1	0.070
Bi3	2*g*	1	1	0.6116(7)	1	0.049
S1	2*g*	1	1	0.8366(05)	0.97(5)	0.013
S2	1*c*	0.5	0.5	1	1	0.063
Cl1	1*d*	0.5	0.5	0.5	1	0.104
O1	4*i*	1	0.5	0.6802(3)	1	0.056

Table S3. The typical bond lengths and bond angles of $Bi_5O_4S_3Cl$ (NPD), $Bi_3O_2S_2Cl$, Bi_2OS_2, $Bi_4O_4S_3$, and $LaOBiS_2$.[1]

	$Bi_5O_4S_3Cl$	$Bi_3O_2S_2Cl$	Bi_2OS_2	$Bi_4O_4S_3$	$LaOBiS_2$
Bi2-O1 (Å)	2.410(5)	2.366(3)	2.350(8)	2.416(10)	—
O1-Bi2-O1 (°)	108.6(3)	112.1(5)	114.8(1)	110.3(3)	—
Bi1-S1 (Å)	2.732(1)	2.819(1)	2.496(7)	2.474(9)	2.476(5)
Bi1-S2/Cl (Å)	2.768(2)	2.776(8)	2.819(9)	2.812(2)	2.880(5)
S1-Bi1-S2 (°)	90	90	96.68(1)	94.62(2)	93.82(1)

Table S4. The corrsponding supercells and optimized structural parameters for $Bi_5O_4S_xCl$ with x = 3, 2.9, 2.857, 2.8, 2.75, and 2.67. The sulfur vacancy is located at S1 site.

x	3	2.9	2.857	2.8	2.75	2.67
Supercell	1*1*1	2*5*1	1*7*1	1*5*1	2*2*1	1*3*1
a	3.9411	7.9041	3.9431	3.9501	7.9457	3.9552
b	3.9411	19.8021	27.9186	19.9805	7.9457	12.0484
c	17.0074	16.9161	16.8793	16.8240	16.7941	16.6858
Band gap (eV)	0.52	metal	metal	metal	metal	metal

Table S5. The corrsponding supercells and optimized structural parameters for $Bi_5O_4S_xCl$ with x = 3, 2.9, 2.857, 2.8, 2.75, and 2.67. The sulfur vacancy is located at S2 site.

x	3	2.9	2.857	2.8	2.75	2.67
Supercell	1*1*1	2*5*1	1*7*1	1*5*1	2*2*1	1*3*1
a	3.9411	7.8663	3.9285	3.9206	7.8395	3.9003
b	3.9411	19.6456	27.4716	19.5970	7.8395	11.7355
c	17.0074	17.0193	17.0303	17.0563	17.0755	17.1142
Band gap (eV)	0.52	metal	metal	metal	metal	metal

Table S6. The relative energy difference per atom for $Bi_5O_4S_xCl$ with the same x = 2.9, 2.857, 2.8, 2.75, and 2.67.

x	2.9	2.857	2.8	2.75	2.67
$\Delta E^{a)}$/atom (meV)	-0.59	-0.91	2.06	13.29	16.58

$^{a)}$ $\Delta E = E(\mathrm{S1}) - E(\mathrm{S2})$, E(S1) and E(S2) are relative energy of $Bi_5O_4S_xCl$ (x = 2.9, 2.857, 2.8, 2.75, and 2.67) when vacancies are located at S1 site and S2 site, respectively.

3. References

[1] a) B. B. Ruan, K. Zhao, Q. G. Mu, B. J. Pan, T. Liu, H. X. Yang, J. Q. Li, G. F. Chen, Z. A. Ren, *J. Am. Chem. Soc.* **2019**, *141*, 3404-3408; b) A. Miura, Y. Mizuguchi, T. Takei, N. Kumada, E. Magome, C. Moriyoshi, Y. Kuroiwa, K. Tadanaga, *Solid State Commun*. **2016**, *227*, 19-22; c) A. Miura, Y. Mizuguchi, T. Sugawara, Y. Wang, T. Takei, N. Kumada, E. Magome, C. Moriyoshi, Y. Kuroiwa, O. Miura, K. Tadanaga, *Inorg. Chem*. **2015**, *54*, 10462-10467.
